# Microbial Community Analysis and Environmental Association in Cave 6 of the Yungang Grottoes

**DOI:** 10.3390/microorganisms13122788

**Published:** 2025-12-07

**Authors:** Shangxiao Qiao, Zeao Wang, Runping Zhang, Yu Wang, Cen Wang, Guoming Gao, Jiao Pan

**Affiliations:** 1 Yungang Academy, Datong 037007, China; qiaoshangxiao115@163.com (S.Q.); zhrp433@126.com (R.Z.); 2 Shanxi Key Laboratory of Grotto Temple Protection and Inheritance, Datong 037007, China; 3 Key Scientific Research Base of the Study of Yungang, State Administration of Cultural Heritage, Datong 037007, China; 4 Key Laboratory of Archaeomaterials and Conservation, Ministry of Education, University of Science and Technology Beijing, Beijing 100083, China; 15026209072@163.com (Z.W.); d202310766@xs.ustb.edu.cn (Y.W.); d202410811@xs.ustb.edu.cn (C.W.); m202411471@xs.ustb.edu.cn (G.G.); 5 Institute for Cultural Heritage and History of Science and Technology, University of Science and Technology Beijing, Beijing 100083, China

**Keywords:** Yungang Grottoes, microbial community, high-throughput sequencing, bioweathering, cultural heritage conservation

## Abstract

The Yungang Grottoes, a World Heritage Site, face biodeterioration risks. This study analyzed microbial communities in five microenvironments within Cave 6 using high-throughput sequencing (16S/18S rRNA). Communities showed high microenvironment specificity. Ascomycota and Proteobacteria dominated fungi and bacteria, respectively. Areas near the lighting window, with high external interaction, showed the highest diversity, while red pigment areas, likely under heavy metal stress, had the lowest diversity. Human-associated microbes (e.g., Escherichia-Shigella, Malassezia) indicated anthropogenic pollution on statue surfaces. Core microbiome and functional prediction (PICRUSt2) suggested high biodegradation risk in dust accumulation and inter-statue areas, enriched with organic-degrading and acid-producing taxa (e.g., Rubrobacter, Cladosporium). Microbial distribution and function were driven by openness, substrate, and human impact. This study identifies key risk zones and informs targeted conservation strategies for the Yungang Grottoes.

## 1. Introduction

The Yungang Grottoes, located on the southern cliff of Wuzhou Mountain west of Datong City, Shanxi Province, China, is one of China’s four major grotto complexes and was inscribed on the UNESCO World Heritage List in 2001. Cave 6 at Yungang was excavated during the reign of Emperor Xiaowen of the Northern Wei Dynasty (471–494 AD) and is a representative cave from the middle period (second phase) of Yungang, completed before the capital moved to Luoyang in 494 AD [1,2]. Cave 6 represents the pinnacle of Northern Wei royal cave art, integrating architectural innovation, religious narrative, and artistic fusion. Its central pillar structure, 37 carved scenes from the Buddha’s life, and Sinicized statues not only showcase the brilliance of 5th-century Buddhist art but also mark a significant turning point in the localization of Buddhism in China [3,4,5]. The carvings and paintings within the cave rely on sandstone and limestone rock mass [6], materials susceptible to various deterioration risks under long-term natural stress and human influence, among which biodeterioration, particularly microbial growth and metabolic activity, has become a non-negligible factor.

In recent years, studies on microbial communities based on molecular biology techniques have provided new perspectives for cultural heritage conservation [7,8]. Compared to traditional culture-dependent methods, high-throughput sequencing enables a comprehensive analysis of the composition and structure of complex microbial communities on relic surfaces [9,10]. By extracting total DNA from samples and sequencing specific gene fragments (such as bacterial 16S rRNA and fungal 18S rRNA), researchers can rapidly and accurately reveal the composition and relative abundance of microbial communities, providing a scientific basis for conservation efforts [11,12]. For example, regarding the stone carvings and steles within the Cultural Landscape of West Lake in Hangzhou, China, high-throughput sequencing was utilized to identify colonizing microbial communities, with *Cyanobacteria* identified as the predominant bacterial phylum [13].

Beyond community identification, understanding the potential biodeterioration mechanisms is equally critical. Recent reviews highlight that cave fungi and bacteria possess immense metabolic capabilities that can lead to structural damage through acid production, biomineralization, and pigment secretion [14,15]. For instance, Barbosa [14] emphasized that cave fungi, driven by resource limitation, evolve unique metabolic pathways to produce bioactive compounds and enzymes that can chemically alter their substrate. Similarly, molecular studies at the Mogao Grottoes have revealed that airborne fungi, particularly *Cladosporium* and *Alternaria* [15], show pronounced seasonal dynamics and are closely linked to environmental fluctuations.

Despite the high artistic value of Cave 6, systematic research on its microbial ecology remains limited [16,17,18]. Previous studies have often treated the cave as a uniform environment, neglecting the micro-environmental heterogeneity that drives microbial community assembly. Understanding these niche-specific variations is crucial, as seen in other subterranean environments where speleothems [19] and rock surfaces act as “microbial arks” [20] preserving distinct bacterial communities. Therefore, decoding these micro-environmental signatures is essential for accurate risk assessment.

Therefore, this study takes Cave 6 of the Yungang Grottoes as the research object. Through targeted sampling strategy and high-throughput sequencing technology, it aims to: (1) analyze the community structure and diversity characteristics of bacteria and fungi in five typical microenvironments; (2) identify key environmental drivers affecting microbial community assembly; (3) assess the distribution of microbial taxa with degradation potential and their functional risks. This research will reveal the relationship between microenvironments and relic deterioration from a microbial ecology perspective, aiming to provide key scientific evidence for risk zoning management and the development of precise preventive conservation strategies for the Yungang Grottoes.

## 2. Materials and Methods

### 2.1. Sampling Site Description and Sample Collection

Cave 6 of the Yungang Grottoes (40°6′36″ N, 113°7′32″ E) is located approximately 16 km west of Datong City on the southern cliff of Wuzhou Mountain. Sampling was conducted in June 2024. The intra-cave environmental temperature was 20 °C, with a relative humidity of 56%. Based on the spatial structure, surface material, and degree of human disturbance within the cave, five types of microenvironments were selected for sampling (Table 1), including: wall junction/cavity areas (Table 1, a, b) (low-disturbance dust accumulation environment), areas around the lighting window (Table 1, c, d) (strong interaction with the outside), red pigment areas (Table 1, e, f) (potentially containing heavy metals like cinnabar), Buddha statue surfaces (Table 1, g, h) (significantly affected by visitor contact), and spaces between Buddha statues (Table 1, i, j) (transition environment). Two biological replicate samples were collected from each environment, totaling 10 samples, labeled YGSK1–YGSK10. Sterile swabs moistened with phosphate buffer were used to repeatedly wipe an area of approximately 10 cm^2^, and the swabs were stored at −80 °C until DNA extraction.

### 2.2. DNA Extraction and High-Throughput Sequencing

Total DNA was extracted from samples using the DNeasy PowerSoil Pro Kit (QIAGEN, Hilden, Germany), strictly following the manufacturer’s instructions. After confirming DNA concentration and purity using NanoDrop, samples were sent to Novogene Co., Ltd. (Beijing, China) for amplicon sequencing. Sequencing was performed on the Illumina NovaSeq 6000 platform, generating PE250 reads. The fungal 18S V4 region was amplified using primers 5′-GCGGTAATTCCAGCTCCAA-3′ and 5′-AATCCRAGAATTT CACCTCT-3′, and the bacterial 16S V4 region was amplified using primers 5′-GTGCCAGCMGCCGCGGTAA-3′ and 5′-GGACTACHVGGGT WTCTAAT-3′.

### 2.3. Bioinformatics and Statistical Analysis

Raw paired-end reads were demultiplexed and primer-trimmed based on unique barcodes. Sequence merging was performed using FLASH (Version 1.2.11) [21], followed by quality filtering with fastp (Version 0.23) [22] to generate high-quality clean tags. Instead of traditional OTU clustering, effective tags were denoised using the DADA2 [23] plugin within the QIIME2 framework (Version 202202) [24] to obtain Amplicon Sequence Variants (ASVs). Taxonomic assignment for both bacterial (16S rRNA) and fungal (18S rRNA) features was conducted using the QIIME2 classifier against the SILVA database (Release 138.1) [25].

Alpha diversity indices, including Chao1, Shannon, Simpson, and Pielou’s evenness, were calculated via QIIME2 to assess community richness and uniformity. For beta diversity, differences in community structure were evaluated using weighted and unweighted UniFrac distances and visualized through Principal Coordinate Analysis (PCoA) using the ade4 [26] and ggplot2 [27] packages in R (Version 4.0.3) [28]. Additionally, the functional potential of the bacterial communities was predicted using PICRUSt2 (Version 2.3.0) [29] by aligning ASV sequences to the reference phylogeny and mapping them to KEGG pathways.

## 3. Results

### 3.1. Microbial Community Composition Structure

#### 3.1.1. Fungal Community

Initial screening of the raw 18S rRNA reads for the Buddha surface group (YGSK7–8) revealed a substantial fraction of non-target sequences annotated as Metazoa (specifically Annelida and Vertebrata). Given that these sampling sites are subject to frequent touching by visitors, these sequences likely represent exogenous biological debris (such as soil dust or skin traces) rather than active microbial colonization. Consequently, these metazoan sequences were filtered out to accurately resolve the fungal community structure.

**Figure 1:**
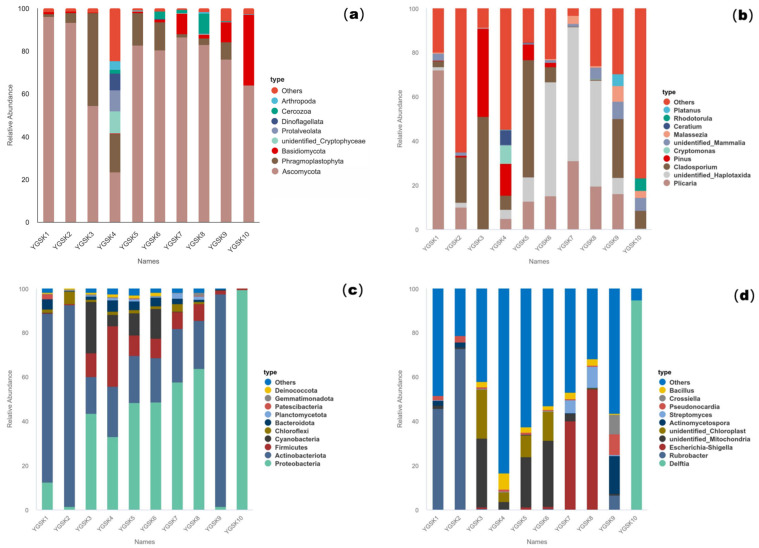
Microbial relative abundance bar charts for samples from Cave 6: (**a**): 18S phylum level; (**b**): 18S genus level; (**c**): 16S phylum level; (**d**): 16S genus level.

After filtering, at the phylum level, Ascomycota was the dominant phylum across all microenvironments (average relative abundance 73.86%), followed by Basidiomycota (5.86%). The wall junction/cavity group (YGSK1–2) was almost exclusively Ascomycota (94.56%), whereas the external interaction group (YGSK3–4) contained a more diverse assemblage of exogenous eukaryotes like Cryptophyta and Dinoflagellata. In the Buddha surface group (YGSK7–8), the corrected profile was primarily composed of Ascomycota and Basidiomycota. At the genus level, the dominant genera were *Plicaria* (18.16%) and *Cladosporium* (17.49%), with significant distribution differences among groups (Figure 1a,b).

#### 3.1.2. Bacterial Community

At the phylum level, Proteobacteria (40.98%) and Actinobacteria (30.05%) were the dominant groups. The wall junction/cavity group was dominated by Actinobacteria (83.76%, with *Rubrobacter* accounting for 59.37%). The proportions of Proteobacteria, Cyanobacteria, and Firmicutes increased in the external interaction and pigment groups. *Escherichia-Shigella* was highly abundant (47.18%) on Buddha surfaces, indicating fecal contamination risk. Dominant bacteria at the genus level differed significantly among groups, reflecting diverse environmental selection pressures (Figure 1c,d).

### 3.2. Alpha Diversity

**Figure 2:**
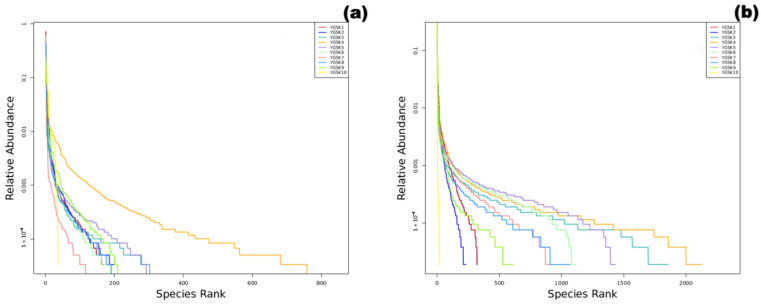
Sample Rank Abundance curves ((**a**): fungi, (**b**): bacteria).

Alpha diversity indices for fungal and bacterial communities are shown in Table 2 and Table 3. The external interaction group (YGSK3 and YGSK4) had the highest species richness (Chao1) and diversity (Shannon). Their Rank Abundance curves (Figure 2) spanned the widest on the *x*-axis and were relatively flat, indicating significantly increased species abundance due to environmental interaction. In contrast, the red pigment area sample (YGSK6) had the lowest diversity, poor evenness, and a high dominance index, indicating strong selective pressure from pigment components on the community. The Buddha surface group (YGSK7 and YGSK8) had lower diversity and poor evenness, indicating the impact of human disturbance on community structure.

### 3.3. Core Microbiome Analysis

To identify core microbial taxa coexisting with high relative abundance across the five microenvironments, particularly focusing on functional groups related to relic deterioration like acid production and organic matter degradation, core microbiome analysis was performed. The screening criteria were: presence in no less than 4 types (i.e., 80%) of microenvironments, with an average relative abundance at the genus level not less than 0.5%.

**Figure 3:**
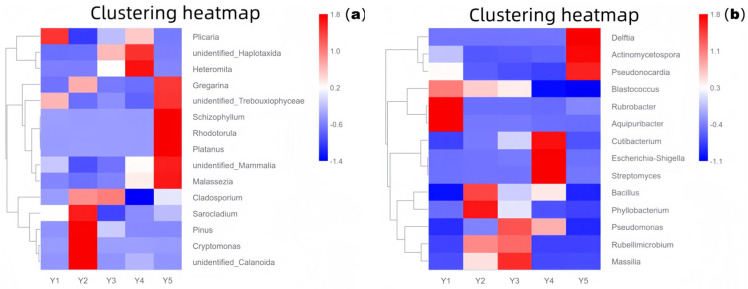
Core microbiome relative abundance heatmap ((**a**): 18S rRNA fungi; (**b**): 16S rRNA bacteria). Y1: Wall junction/cavity group; Y2: External interaction group; Y3: Pigment group; Y4: Buddha surface group; Y5: Between Buddha group.

As shown in Figure 3 (a: fungal community based on 18S rRNA; b: bacterial community based on 16S rRNA), the composition of core microbial taxa differed significantly among the five microenvironments. Group Y1 (wall junction/cavity) was dominated by *Rubrobacter*, common in stone environments, known for tolerance to desiccation and UV, and capable of metabolizing various organic acids, suggesting a potential role in stone bioweathering, consistent with its dusty, low-disturbance environment. Group Y2 (external interaction) had the highest overall species richness, with relatively high proportions of *Pinus* and *Cryptomonas*, indicating significant external input (e.g., air, pollen, algae), consistent with the high airflow near the lighting window. In Group Y3 (pigment), the relative abundance of *Cladosporium* was significantly high; this fungal genus is widely involved in organic matter degradation, can produce pigments and various extracellular enzymes, and may be closely related to the decomposition of organic binders and fading processes in the pigment layer. In Group Y4 (Buddha surface), *Escherichia-Shigella* was extremely prominent, strongly indicating fecal contamination introduced by human contact, requiring high attention to its potential public health risk and biochemical erosion of relic surfaces. In Group Y5 (between Buddhas), *Pseudonocardia* was relatively abundant; these actinomycetes are known for their ability to degrade complex organic matter and may participate in the decomposition of organic material accumulated between statues, warranting vigilance regarding their potential biodeterioration effect.

### 3.4. Unique Species Analysis by Microenvironment

To quantify the number of unique species in the five microenvironments and visually present the shaping effect of microenvironmental selection pressure on microbial community structure, unique species analysis was performed.

**Figure 4:**
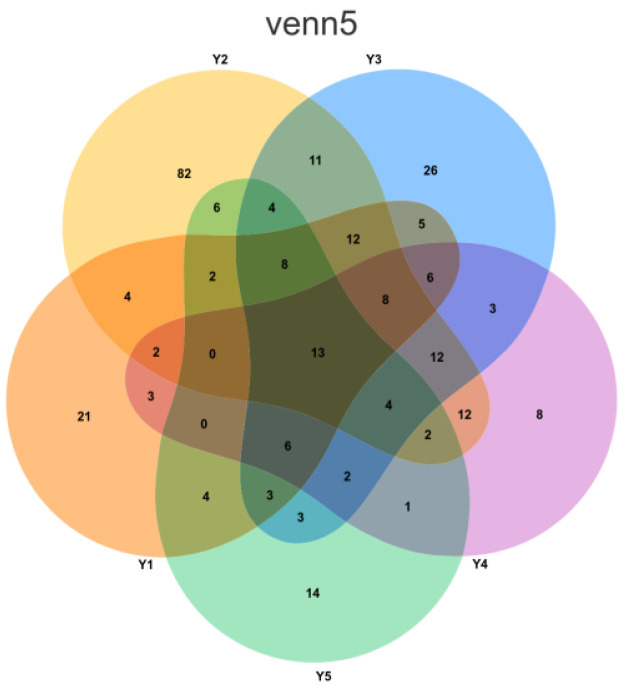
Venn diagram illustrating the distribution of Amplicon Sequence Variants (ASVs) across the five sampling groups. Each group is represented by a distinct color: orange for Y1 (Wall junction/cavity), yellow for Y2 (External interaction), blue for Y3 (Pigment), purple for Y4 (Buddha surface), and green for Y5 (Between Buddha). The numerical values within the diagram sections denote the count of ASVs that are either unique to a single group or shared among multiple groups.

Based on Venn diagram analysis (Figure 4), the number of unique species varied significantly among the five microenvironment groups. Group Y2 (external interaction, samples YGSK3 and YGSK4) had the highest number of unique species, reaching 82. This group, located near the lighting window with strong air circulation and frequent interaction with the outside environment, easily receives and temporarily harbors various exogenous microorganisms (e.g., airborne dust, plant pollen, visitor-borne microbes), resulting in higher unique species diversity. In contrast, Group Y4 (Buddha surface, samples YGSK7 and YGSK8) had the lowest number of unique species, only 8. This area is directly exposed to visitor contact, subject to continuous disturbances like touching. This high-frequency disturbance likely inhibits the colonization and succession of specific microbial communities, leading to a significantly lower number of unique species.

### 3.5. Beta Diversity Analysis

To analyze the overall structural differences of bacterial (16S rRNA gene) and fungal (18S rRNA gene) communities in different microenvironments of Cave 6, Principal Coordinate Analysis (PCoA) was performed based on the Bray–Curtis distance algorithm (emphasizing contributions from dominant species via relative abundance) to visualize community similarity and differentiation patterns among samples.

(1) Fungal Community Structure Characteristics

**Figure 5:**
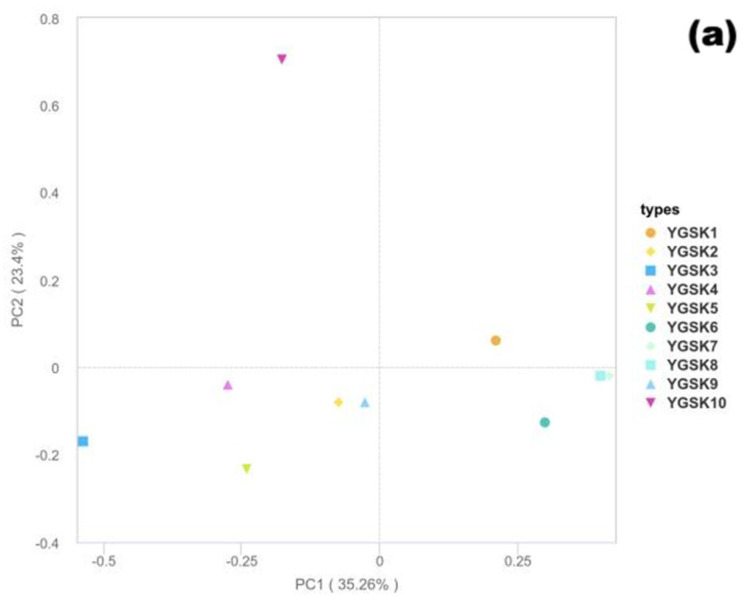

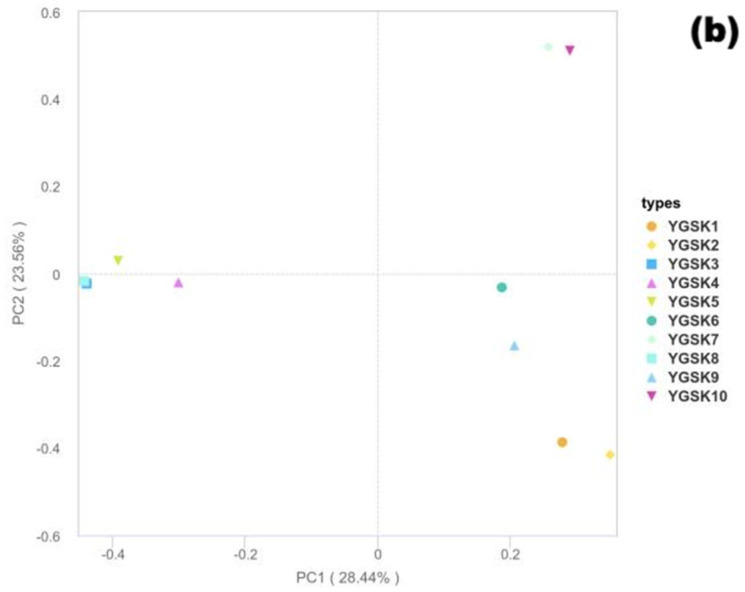
Bray–Curtis PCoA results ((**a**): 18S rRNA fungi; (**b**): 16S rRNA bacteria).

The PCoA results based on the 18S rRNA gene (Figure 5a) showed that PC1 and PC2 explained 35.26% and 23.40% of the community variation, respectively, with a cumulative contribution of 58.7%, indicating high explanatory power for fungal community structure differences. Sample distribution showed microenvironment aggregation trends: YGSK7 and YGSK8 (Buddha surface group) clustered relatively together near the positive PC1 axis and around zero on PC2, suggesting strong selective effects of this environment on the fungal community; YGSK10 (between Buddha group) was distributed independently at the positive end of PC2, indicating the specificity of its community structure.

(2) Bacterial Community Structure Characteristics

In the PCoA analysis based on the 16S rRNA gene (Figure 5b), PC1 and PC2 explained 28.44% and 23.56% of the variation, respectively, accounting for 51.99% of the community differences cumulatively. Sample distribution showed a clear microenvironment-driven pattern: YGSK1 and YGSK2 (wall junction/cavity group) clustered in the positive PC1 and negative PC2 region; YGSK7 and YGSK10 (Buddha surface and between Buddha groups) clustered in the positive PC1 and positive PC2 region, reflecting the significant influence of different microenvironments on bacterial community assembly. The differences in PCoA distribution between bacterial and fungal communities reflect the “differential selection effect” of microenvironments on different microbial groups.

(3) Differences in Community Differentiation Mechanisms

Bacterial communities were more easily driven by heterogeneity factors such as organic carbon source types and human disturbance intensity in the microenvironment, showing higher community dispersion; whereas fungal communities showed stronger adaptability to specific substrates (e.g., organic components of pigments, human secretions), leading to more convergent community structures in certain microenvironments (e.g., Buddha surfaces). This difference highlights the differential selection pressure of microenvironmental factors on different microbial groups.

### 3.6. PICRUSt2 Functional Prediction (KEGG Pathways Focused on Deterioration-Related Functions

**Figure 6:**
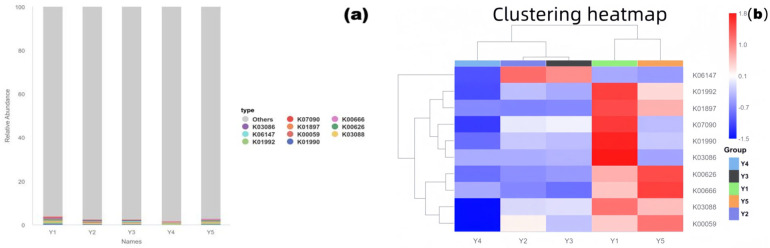
Community functional prediction results ((**a**): KEGG pathway stacked bar chart; (**b**): KO abundance clustered heatmap). Y1: Wall junction/cavity group; Y2: External interaction group; Y3: Pigment group; Y4: Buddha surface group; Y5: Between Buddha group.

To assess the potential biodeterioration risk of microbial communities to the grotto relics, the PICRUSt2 tool was used to predict community functional composition (KEGG Orthologs, KOs) based on 16S rRNA gene sequences and the Greengenes database. Stacked bar charts (Figure 6a) and clustered heatmaps (Figure 6b) were used to reveal the differentiation and enrichment characteristics of functional profiles in different microenvironments.

(1) Functional Composition Structure

The stacked bar chart showed that core KOs like K01990, K03088, and K00059 were present in all microenvironments, constituting conservative functional profiles such as basic metabolism and substance transport. Simultaneously, the functional composition of each group also showed significant environmental specificity.

(2) Functional Clustering and Enrichment of Deterioration-Related Pathways

The clustered heatmap showed that samples clustered into three groups based on functional profile similarity: Y1 with Y5, Y2 with Y3, and Y4 alone, indicating that microbial functional potential is microenvironment-specific. Notably, KOs related to organic matter degradation (e.g., K01897, K07090, annotated as polysaccharide-degrading enzymes) were significantly enriched in groups Y1 and Y5 (red areas in the figure), indicating that microorganisms in these areas have a strong capacity for degrading organic matter, potentially directly participating in the decomposition of organic components like pigment binders and dust, exacerbating relic deterioration. Highly abundant KOs in group Y4 (Buddha surface) were mostly related to stress response (e.g., K06147, oxidative stress response), reflecting the survival strategies of its community adapting to high-frequency human disturbance and potential stress pressures.

(3) Implications for Heritage Conservation

Areas Y1 and Y5 require focused prevention and control of biodeterioration caused by organic matter degradation. Regular removal of dust and exogenous organic matter is recommended to cut off microbial metabolic substrates. Active acid production-related metabolic pathways (e.g., K00059) were also detected in area Y5, necessitating vigilance against erosion of stone surfaces by acidic metabolites; therefore, enhanced environmental monitoring and physical removal of acidic deposits are advised to mitigate the risk. The unique functional profile of group Y4, with its antioxidant mechanisms, is related to its low organic matter and high disturbance environment. Further validation of actual functional gene expression using metagenomics is needed to accurately assess its deterioration risk.

## 4. Discussion

This study utilized high-throughput sequencing technology to systematically analyze the microbial community structure, diversity, and potential functions in five typical microenvironments within Cave 6 of the Yungang Grottoes. It revealed the key driving role of microenvironment heterogeneity in microbial community assembly and assessed the potential biodeterioration risks to the grotto relics.

### 4.1. Association Between Microbial Community Structure and Environmental Factors

In terms of community structure, Ascomycota was the absolutely dominant group among fungi. This phylum generally has strong environmental adaptability and the ability to degrade organic matter, characteristics that position them as primary pioneer colonizers on lithic substrates. This finding aligns with a recent global meta-analysis [30], which identified Ascomycota as a persistent threat to stone monuments due to their hyphal penetration capabilities and organic acid secretion. Similarly, samples from the Cave Church of Sts. Peter and Paul in Serbia [31] and the Mogao Grottoes [32] were also dominated by Ascomycota. Crucially, as Sterflinger (2010) [33] and recent updates by Geweely (2023) [8] highlight, the dominance of black meristematic fungi within this phylum represents a specific adaptation to extremes of radiation and desiccation, typical of open grotto environments.

It is worth noting that while non-microbial sequences were removed for diversity analysis, the detection of high-abundance Metazoa DNA (e.g., Annelida from soil dust) in the raw data of the Buddha surface group (YGSK7, YGSK8) serves as a distinctive anthropogenic footprint. Unlike aquatic biofilm formation, this accumulation likely results from the mechanical transfer of soil particles and environmental debris via frequent tourist contact. Chen et al. (2020) [34] disturbance demonstrate that human activity directly reshapes microbial communities through mechanical transfer of particles and introduction of exogenous organic matter. Human footfall and contact represent significant vectors for particle and microbial transfer.

Proteobacteria and Actinobacteria were widely distributed in the bacterial community, with Actinobacteria absolutely dominant (83.76%) in the wall junction/cavity group (YGSK1, YGSK2). This group is known for its ability to degrade complex organic matter, produce spores, and pigments [35]. Its enrichment in dust accumulation areas is consistent with findings by Mohammadipanah et al. (2022) [36], who noted that Actinobacteria, particularly *Rubrobacter*, serve as bio-indicators of salt efflorescence and desiccation stress in arid grotto environments.

Particularly, the external interaction group (YGSK3, YGSK4) showed higher community complexity and input of exogenous taxa, likely introduced through the lighting window via air or rainwater. This suggests that environments connected to the outside are more susceptible to invasion by environmental microorganisms. This result indicates that microbial dynamics in such areas require special attention, a view supported by Martin-Pozas et al. (2024) [37] who note that culturally important sites with external connections, such as caves or exhibition halls with lighting openings, are more vulnerable to colonization and invasion by environmental microorganisms. Furthermore, the micro-environmental heterogeneity observed in Cave 6 mirrors mechanisms recently elucidated in soil ecology. Simon et al. (2024) [38] demonstrated that millimeter-sized soil aggregates function as distinct microhabitats, creating stable niches that harbor specific microbial communities different from the surrounding bulk soil. Analogously, the dust deposits in the wall junctions and cavities (Groups Y1, Y2) likely function as “pseudo-aggregates,” providing a physical scaffold and a stable, oligotrophic niche that selectively favors desiccation-tolerant taxa like *Rubrobacter.* This suggests that the accumulation of dust is not merely a physical deposition process but an ecological driver that shapes community assembly at the micro-scale. Additionally, the environmental gradient from the aerated lighting window to the sheltered pigment layers plays a decisive role in shaping community turnover. This aligns with observations by Li et al. (2017) [39] on the Tibetan Plateau, where variations in soil water balance and nutrient stoichiometry were identified as primary drivers of microbial community structure across landscape gradients. In Cave 6, the steep gradient of air circulation and humidity acts as a similar deterministic filter, fundamentally altering community composition over short spatial distances.

The strong differences we observed between the microhabitats in Cave 6 show that the local environment acts as a strict filter for the microbial community. We cannot view the grotto as a single, uniform ecosystem. It behaves more like a collection of separate small islands. Local conditions in each specific spot dictate exactly which microbes can survive there. This pattern aligns with findings from other heritage sites. For instance, Vagelas et al. described certain cave formations as “microbial arks” that preserve distinct bacteria because of their unique mineral makeup (155) [19]. Similarly, research on a Portuguese stone convent proved that microbes do not settle on rocks randomly [9]. The physical and chemical traits of the stone surface drive this process.

Our results in Cave 6 confirm this filtering effect. The area near the lighting window receives fresh air and supports a diverse mix of species. In contrast, the pigment areas likely contain heavy metals that create chemical stress. This stress limits the community to a few adapted types. This creates isolated pockets of life within the same cave. Therefore, conservation efforts cannot use a single approach for the whole site. We must customize our protection strategies to fit the specific risks found in each small area.

### 4.2. Potential Causes of Alpha Diversity Differences

Alpha diversity analysis showed that the external interaction group (YGSK3 and YGSK4) had significantly higher diversity indices, indicating high habitat complexity. Conversely, YGSK6 (red pigment area) had extremely low diversity, likely related to heavy metal ions (e.g., mercury in cinnabar). This strong environmental selection pressure limits the growth of most microorganisms. This is consistent with the findings of Ma et al. (2015) [32] on the Mogao Grottoes murals, where heavy metal pigments (lead and mercury) were found to significantly reduce microbial diversity while selecting for resistant genera capable of biotransforming these toxins.

The Buddha surface group (YGSK7, YGSK8) exhibited a unique “contact-driven” profile. The co-occurrence of exogenous metazoan DNA and high relative abundances of human-associated bacteria (e.g., *Escherichia-Shigella*) provides strong evidence of human-mediated transport. Visitors appear to act as vectors, transferring soil residues and skin flora to the statues. This aligns with the “Human-Microbe-Relic” interaction model proposed by Wang et al. (2010) [40], which quantified how skin microbiome dispersion significantly alters the ecological networks of heritage sites during peak tourism seasons. The anthropogenic alteration of the Buddha surface can be conceptualized as a localized “eutrophication” event on the lithic substrate. Recent research on bacterioplankton in aquatic ecosystems by Kubera et al. (2025) [41] highlighted that trophic status is a robust predictor of bacterial community structure and metabolic potential. By analogy, the deposition of skin oils and sweat creates nutrient-rich “islands” on the oligotrophic stone surface. These anthropogenic nutrient inputs likely lower the metabolic constraints for colonization, supporting higher microbial activity and distinct assemblies compared to the pristine rock. Moreover, the physical nature of visitor contact—occurring as discrete, random touches—creates a fragmented landscape for microbial growth. Mant et al. (2025) [42] revealed that in fragmented habitats, the size and distribution of micro-patches significantly dictate population dynamics through stochastic seeding processes. On the statues, the patchy distribution of anthropogenic nutrients likely prevents competitive exclusion by indigenous litholytic microbes, allowing opportunistic human-associated pathogens to persist in these spatial refugia, thereby complicating the biodeterioration risk profile.

### 4.3. Biodeterioration Risk Assessment and Conservation Implications

In terms of biodeterioration potential, the detection of genera such as *Cladosporium*, *Rubrobacter*, and *Streptomyces*, at high abundances requires attention. For example, the high proportion of Cladosporium in the red pigment areas may suggest its potential role in decomposing the pigment layer. Recent metabolomic analyses by Pyzik et al. (2021) [43] have confirmed that Cladosporium species can solubilize various mineral pigments through the excretion of gluconic and oxalic acids, posing a severe risk of chromatic alteration.

On the other hand, although YGSK4 has high diversity the variety of exogenous microorganisms it contains could also rapidly proliferate under suitable temperature and humidity conditions. It is recommended to strengthen microbial monitoring in areas like the lighting window. As Liu et al. (2020) [30] argue, moving from reactive treatment to preventive monitoring of these “invasion gateways” is critical for sustainable conservation.

### 4.4. Beta Diversity Analysis

Beta diversity analysis results indicated that bacterial community structure is more easily driven by microenvironment resource heterogeneity (e.g., differences in carbon source types), while fungal communities show stronger specific adaptation to substrate characteristics (e.g., organic components of pigments, human secretions). This difference reflects the different ecological strategies employed by bacteria and fungi in response to environmental selection pressures.

### 4.5. Functional Prediction

Functional prediction results further emphasized the close relationship between microbial function and microenvironment adaptation: the significant enrichment of genes for polysaccharide-degrading enzymes (e.g., K01897, K07090) and acid production-related genes (K00059) in areas Y1 (wall junction/cavity) and Y5 (between Buddhas) indicates that microbial communities in these areas have strong potential for organic matter degradation, potentially directly involved in the decomposition of organic binders and acidification of stone surfaces; microorganisms in area Y4 (Buddha surface) highly expressed stress response-related genes, reflecting their survival adaptation mechanisms to high-frequency human disturbance and low-nutrient environments. These findings not only provide new perspectives for understanding the ecological functions of grotto microorganisms but also offer a theoretical basis for formulating targeted conservation strategies.

Based on our microbial risk map, we propose specific conservation actions. The wall junctions and gaps trap dust which feeds microbes. Therefore, conservation teams should prioritize a targeted cleaning schedule. Eliminating this nutrient supply is the most effective way to starve harmful microbes, a strategy validated by Fidanza and Caneva (2019) [44], who demonstrated that dry cleaning significantly reduces recolonization rates compared to biocide application alone.

The situation near the lighting window requires a different approach. This area serves as a major entry point for outside spores and pollen. To block this biological input, we suggest installing breathable air filters or fine mesh screens on the windows. Ilieș et al. (2022) [45] prove this physical barrier will effectively cut off the route for invading species. For the Buddha statues that people can reach, our data confirms severe contamination from visitors. The high load of human bacteria proves that current restrictions are insufficient. We strongly recommend setting up rigid physical barriers, such as glass partitions or fences, to stop direct contact. Staff should also perform regular, gentle cleaning on these accessible surfaces to remove the skin oils and sweat that visitors leave behind. Turrini et al. (2022) [46] prove this practice serves to prevent the deposition of these anthropogenic organic nutrients, effectively starving the harmful microorganisms by depriving them of their food source. These tailored strategies directly address the unique biological threats we identified in each section.

## 5. Conclusions

Through systematic analysis of microbial communities in different microenvironments of Cave 6 at the Yungang Grottoes, this study comprehensively analyzed the community structure, diversity, and functional potential characteristics of fungi and bacteria for the first time, yielding the following main conclusions:

(1) The microbial community structure within the cave exhibited significant microenvironment heterogeneity. Ascomycota and Proteobacteria were the overall dominant phyla for fungi and bacteria, respectively, but genus-level composition varied significantly across different microenvironments, reflecting strong filtering effects of niche conditions on microbial distribution.
(2) Microbial diversity was jointly influenced by environmental openness and external stress factors. Areas with frequent external interaction (e.g., around the lighting window) had the highest species diversity, while the red pigment area, inhibited by heavy metals (e.g., mercury in cinnabar), showed significantly reduced diversity.
(3) Significant signals of human-derived microorganisms, such as *Malassezia* and *Escherichia-Shigella*, were detected on Buddha surface communities, clearly indicating biological pollution introduced by visitor contact. Their potential public health risks and biochemical erosion of relic surfaces require high attention.
(4) Multiple microbial taxa with known degradation functions (e.g., *Cladosporium*, *Rubrobacter*, and *Streptomyces*) were significantly enriched in dust accumulation areas (wall junctions/cavities) and open areas (around the lighting window), identifying these as high-risk zones for biodeterioration that should be key targets for future monitoring and protection.
(5) Bacteria and fungi exhibited distinctly different response strategies to environmental factors: bacterial community structure was more susceptible to microenvironment resource heterogeneity and human disturbance intensity, while fungal communities showed stronger substrate-specific adaptation to organic substrate types (e.g., pigment binders, human secretions).
(6) Functional prediction analysis further indicated that microbial communities in dust accumulation areas and areas between Buddha statues were enriched with genes related to organic matter degradation and acid production metabolism, predicting a high potential risk of biodeterioration in these areas. The interventions include removal of dust deposits, installation of breathable air filters or fine mesh screens on windows, and placement of glass barriers or railings for accessible Buddha statues.
(7) This study, from the perspectives of microbial community structure, ecological function, and potential risk, clarified the driving mechanisms of microenvironmental factors on the assembly and functional selection of grotto microbial communities. The research results can provide theoretical basis and practical reference for the scientific conservation and precise prevention and control of the Yungang Grottoes and other similar cultural heritage sites.

## 6. Research Limitations and Outlook

We recognize that this study has certain constraints, most notably the limited number of biological samples. Our dataset consisted of ten samples with two replicates per microenvironment, a decision strictly dictated by the conservation priorities of the Yungang Grottoes which necessitate minimal physical intervention to preserve the fragile relic surfaces. Since the sampling was conducted exclusively in June, the current results do not capture seasonal fluctuations or the potential impact of tourist variability over time.

Furthermore, the absence of simultaneous monitoring for specific microenvironmental parameters—such as light intensity, humidity, and organic matter content—limits our ability to fully correlate microbial community structure with microclimatic conditions. We also acknowledge that functional predictions based on 16S rRNA gene sequences are indicative rather than definitive, requiring further validation through metagenomic or metabolomic technologies.

Future investigations will aim to bridge these gaps by implementing a long-term monitoring strategy that spans different seasons. By integrating multi-omics methods with continuous environmental monitoring, we hope to deeply analyze the dynamic processes of microbial functional activities and their specific deterioration mechanisms. This holistic approach will ultimately provide more precise and effective scientific strategies for the preventive conservation of the Yungang Grottoes and other stone cultural relics.

## Figures and Tables

**Table 1:** Grouping and sample correspondence table.

Group	Sampled Images	Sample	Sampling Location	Environmental Characteristics
Wall Junction/Hole Group	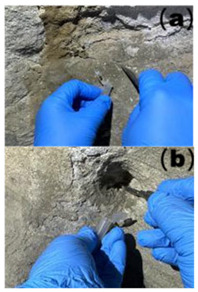	YGSK1, YGSK2	(a): YGSK1 East wall, at the junction between the Ritual Buddha Layer and the North wall; (b): YGSK2 East wall, in a hole on the right side outside the niche of the first standing Buddha from north to south;	Low-disturbance deposition area
External Interaction Group	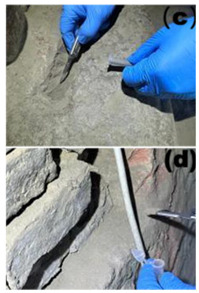	YGSK3, YGSK4	(c): YGSK3 Mingchuang (window), right-side groove; (d): YGSK4 Mingchuang (window), lower wall surface, lower left corner;	Air circulation area
Pigment Group	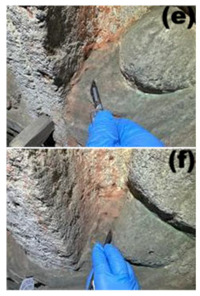	YGSK5, YGSK6	(e): YGSK5 East wall, red pigment layer of the third small seated Buddha from south to north; (f): YGSK6 South wall, red pigment layer of the fourth small seated Buddha from west to east;	Red pigment area
Buddha Statue Surface Group	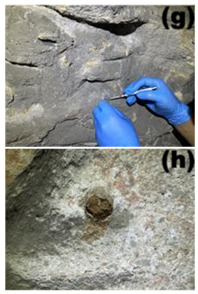	YGSK7, YGSK8	(g): YGSK7 West wall, leg portion of the first small seated Buddha from north to south; (h): YGSK8 North wall, circular hole on the abdomen of the first Vajrapani (guardian figure);	Buddha statue surface area
Inter-Buddha Area Group	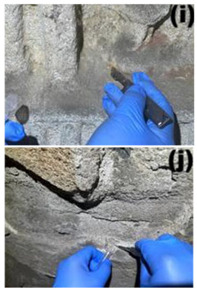	YGSK9, YGSK10	(i): YGSK9 West wall, between the first and second small seated Buddhas from south to north; (j): YGSK10 North wall, between the first and second small seated Buddhas from west to east.	Area between adjacent Buddha statues

**Table 2:** Alpha diversity analysis of fungal communities.

Sample_Name	Chao1	Dominance	Goods_Coverage	Observed_Features	Pielou_e	Shannon	Simpson
YGSK1	170	0.523	1	170	0.292	2.16	0.477
YGSK2	198	0.237	1	198	0.422	3.218	0.763
YGSK3	307.677	0.381	1	307	0.276	2.283	0.619
YGSK4	780.444	0.034	1	774	0.687	6.596	0.966
YGSK5	302.214	0.305	1	299	0.37	3.045	0.695
YGSK6	36	0.095	1	36	0.761	3.932	0.905
YGSK7	125.4	0.453	1	123	0.24	1.666	0.547
YGSK8	197.462	0.296	1	194	0.37	2.815	0.704
YGSK9	211.111	0.114	1	211	0.532	4.105	0.886
YGSK10	203.118	0.283	1	201	0.361	2.76	0.717

**Table 3:** Alpha diversity analysis of bacterial communities.

Sample_Name	Chao1	Observed_Features	Dominance	Goods_Coverage	Pielou_e	Shannon	Simpson
YGSK1	326	323	0.072	1	0.644	5.365	0.928
YGSK2	232.161	231	0.145	1	0.525	4.126	0.855
YGSK3	1863.63	1857	0.115	0.999	0.594	6.449	0.885
YGSK4	2151.58	2128	0.011	0.998	0.777	8.592	0.989
YGSK5	1441.21	1433	0.039	0.999	0.757	7.939	0.961
YGSK6	21.5	21	0.902	1	0.079	0.346	0.098
YGSK7	926.774	918	0.124	0.999	0.587	5.777	0.876
YGSK8	1129.65	1112	0.085	0.999	0.674	6.824	0.915
YGSK9	632.203	612	0.125	0.999	0.487	4.505	0.875
YGSK10	1123.76	1069	0.207	0.998	0.484	4.868	0.793

## Data Availability

The original data presented in the study are openly available in the National Center for Biotechnology Information (NCBI) BioProject database at https://www.ncbi.nlm.nih.gov/bioproject/PRJNA1345260, under the accession number PRJNA1345260 (accessed on 14 October 2025).

## References

[1] Guo H., Bai B. (2024). Cultural inheritance and application of Yungang Grottoes statues in art education. Arts Educ..

[2] Ozawa M. A Study on the Creation of the Standing Tathagata Buddha in the Upper Niches of Cave 6 at Yungang Grottoes. Proceedings of the 2005 Yungang International Academic Symposium.

[3] Wei Z., Ma M.Y. (2022). Early Grottoes in Hexi from the Perspective of Northern China—Part 2 of the Study on Early Hexi Grottoes. Dunhuang Res..

[4] Huang P. (2025). An Archaeological Study of Northern Wei Buddhist Temples at the Yungang Grottoes. Huaxia Archaeol..

[5] Wang Y.K. (2019). Analysis of the Image Composition in Caves 5 and 6 at Datong Yungang. Dunhuang Res..

[6] Liu R.Z., Zhang B.J., Zhang H., Shi M.F. (2011). Deterioration of Yungang Grottoes: Diagnosis and research. J. Cult. Herit..

[7] Sanmartín P., DeAraujo A., Vasanthakumar A. (2018). Melding the old with the new: Trends in methods used to identify, monitor, and control microorganisms on cultural heritage materials. Microb. Ecol..

[8] Geweely N.S. (2023). New frontiers review of some recent conservation techniques of organic and inorganic archaeological artefacts against microbial deterioration. Front. Microbiol..

[9] Rosado T., Dias L., Lança M., Nogueira C., Santos R., Martins M.R., Candeias A., Mirão J., Caldeira A.T. (2020). Assessment of microbiota present on a Portuguese historical stone convent using high-throughput sequencing approaches. MicrobiologyOpen.

[10] Perini N., Mercuri F., Orlanducci S., Thaller M.C., Migliore L. (2020). The integration of metagenomics and chemical physical techniques biodecoded the buried traces of the biodeteriogens of parchment purple spots. Front. Microbiol..

[11] Deiner K., Bik H.M., Mächler E., Seymour M., Lacoursière-Roussel A., Altermatt F., Creer S., Bista I., Lodge D.M., de Vere N. (2017). Environmental DNA metabarcoding: Transforming how we survey animal and plant communities. Mol. Ecol..

[12] Shumskaya M., Lorusso N., Patel U., Leigh M., Somervuo P., Schigel D. (2023). MycoPins: A metabarcoding-based method to monitor fungal colonization of fine woody debris. MycoKeys.

[13] Li Q., Zhang B., He Z., Yang X. (2016). Distribution and diversity of bacteria and fungi colonization in stone monuments analyzed by high-throughput sequencing. PLoS ONE.

[14] Barbosa R.N., Felipe M.T.C., Silva L.F., Silva E.A., Silva S.A., Herculano P.N., Prazeres J.F.S.A., Lima J.M.S., Bezerra J.D.P., Moreira K.A. (2025). A Review of the Biotechnological Potential of Cave Fungi: A Toolbox for the Future. J. Fungi.

[15] Wang W., Ma X., Ma Y., Mao L., Wu F., Ma X., An L., Feng H. (2011). Molecular characterization of airborne fungi in caves of the Mogao Grottoes, Dunhuang, China. Int. Biodeterior. Biodegrad..

[16] Zhu X., Jie B. (2018). Analysis on the environment of cultural relic as tourist attraction—Take Yungang Grottoes as an example. IOP Conf. Ser. Earth Environ. Sci..

[17] Sun B. (2025). Archaeological and Craftsmanship Exploration of the Cave Wall Structure between Cave 5 and Cave 6 of Yungang Grottoes. Yungang Res..

[18] Qian H. (2014). Research on the Spatial Design of Datong Yungang Grottoes. Master’s Thesis.

[19] Vagelas I., Reizopoulou A., Exadactylos A., Madesis P., Karapetsi L., Michail G. (2023). Stalactites core prospect as environmental “microbial ark”: The Actinomycetota diversity paradigm, first reported from a Greek cave. Pol. J. Microbiol..

[20] Michail G., Karapetsi L., Madesis P., Reizopoulou A., Vagelas I. (2021). Metataxonomic analysis of bacteria entrapped in a stalactite’s core and their possible environmental origins. Microorganisms.

[21] Magoč T., Salzberg S.L. (2011). FLASH: Fast length adjustment of short reads to improve genome assemblies. Bioinformatics.

[22] Chen S., Zhou Y., Chen Y., Gu J. (2018). fastp: An ultra-fast all-in-one FASTQ preprocessor. Bioinformatics.

[23] Callahan B.J., Mcmurdie P.J., Rosen M.J., Han A.W., Johnson A.J.A., Holmes S.P. (2016). DADA2: High-resolution sample inference from Illumina amplicon data. Nat. Methods.

[24] Bolyen E., Rideout J.R., Dillon M.R., Bokulich N.A., Abnet C.C., Al-Ghalith G.A., Alexander H., Alm E.J., Arumugam M., Asnicar F. (2019). Reproducible, interactive, scalable and extensible microbiome data science using QIIME 2. Nat. Biotechnol..

[25] Quast C., Pruesse E., Yilmaz P., Gerken J., Schweer T., Yarza P., Peplies J., Glöckner F.O. (2013). The SILVA ribosomal RNA gene database project: Improved data processing and web-based tools. Nucleic Acids Res..

[26] Dray S., Dufour A.B. (2007). The ade4 package: Implementing the duality diagram for ecologists. J. Stat. Softw..

[27] Wickham H. (2016). Data analysis. ggplot2: Elegant Graphics for Data Analysis.

[28] R Core Team (2016). R: A Language and Environment for Statistical Computing.

[29] Douglas G.M., Maffei V.J., Zaneveld J.R., Yurgel S.N., Brown J.R., Taylor C.M., Huttenhower C., Langille M.G.I. (2020). PICRUSt2 for prediction of metagenome functions. Nat. Biotechnol..

[30] Liu X., Koestler R.J., Warscheid T., Katayama Y., Gu J.-D. (2020). Microbial deterioration and sustainable conservation of stone monuments and buildings. Nat. Sustain..

[31] Grbić M.L., Dimkić I., Savković Ž., Stupar M., Knežević A., Jelikić A., Unković N. (2022). Mycobiome diversity of the cave church of sts. Peter and Paul in Serbia—Risk assessment implication for the conservation of rare cavern habitat housing a peculiar fresco painting. J. Fungi.

[32] Ma Y., Zhang H., Du Y., Tian T., Xiang T., Liu X., Wu F., An L., Wang W., Gu J.-D. (2015). The community distribution of bacteria and fungi on ancient wall paintings of the Mogao Grottoes. Sci. Rep..

[33] Sterflinger K. (2010). Fungi: Their role in deterioration of cultural heritage. Fungal Biol. Rev..

[34] Chen Y., Kuang J., Wang P., Shu W., Barberán A. (2020). Associations between human impacts and forest soil microbial communities. Elem. Sci. Anthr..

[35] Sáiz-Jiménez C. (1993). Deposition of airborne organic pollutants on historic buildings. Atmos. Environ. Part B Urban Atmos..

[36] Mohammadipanah F., Wink J. (2016). Actinobacteria from arid and desert habitats: Diversity and biological activity. Front. Microbiol..

[37] Martin-Pozas T., Fernandez-Cortes A., Cuezva S., Jurado V., Gonzalez-Pimentel J.L., Hermosin B., Ontañon R., Arias P., Cañaveras J.C., Sanchez-Moral S. (2024). Microclimate, airborne particles, and microbiological monitoring protocol for conservation of rock-art caves: The case of the world-heritage site La Garma cave (Spain). J. Environ. Manag..

[38] Simon E., Guseva K., Darcy S., Alteio L., Pjevac P., Schmidt H., Jenab K., Ranits C., Kaiser C. (2024). Distinct microbial communities are linked to organic matter properties in millimetre-sized soil aggregates. ISME J..

[39] Li Y., Adams J., Shi Y., Wang H., He J.-S., Chu H. (2017). Distinct Soil Microbial Communities in habitats of differing soil water balance on the Tibetan Plateau. Sci. Rep..

[40] Wang W., Ma X., Ma Y., Mao L., Wu F., Ma X., An L., Feng H. (2010). Seasonal dynamics of airborne fungi in different caves of the Mogao Grottoes, Dunhuang, China. Int. Biodeterior. Biodegrad..

[41] Kubera Ł., Kalwasińska A., Perliński P. (2025). Assessment of the Structure and Activity of Bacterial Communities as a Tandem Tool for Estimating the Ecological Condition of Lakes in Poland: Assessment of the Structure and Activity of Bacterial Communities. Ecosystems.

[42] Mant D., Orevi T., Kashtan N. (2025). Impact of micro-habitat fragmentation on microbial population growth dynamics. ISME J..

[43] Pyzik A., Ciuchcinski K., Dziurzynski M., Dziewit L. (2021). The bad and the good—Microorganisms in cultural heritage environments—An update on biodeterioration and biotreatment approaches. Materials.

[44] Fidanza M.R., Caneva G. (2019). Natural biocides for the conservation of stone cultural heritage: A review. J. Cult. Herit..

[45] Ilieș D.C., Safarov B., Caciora T., Ilieș A., Grama V., Ilies G., Huniadi A., Zharas B., Hodor N., Sandor M. (2022). Museal indoor air quality and public health: An integrated approach for exhibits preservation and ensuring human health. Sustainability.

[46] Turrini P., Chebbi A., Riggio F.P., Visca P. (2024). The geomicrobiology of limestone, sulfuric acid speleogenetic, and volcanic caves: Basic concepts and future perspectives. Front. Microbiol..

